# Supplementary material for: The interactome of intact mitochondria by cross-linking mass spectrometry provides evidence for coexisting respiratory supercomplexes
Source: Mol Cell Proteomics. 2017 Dec 8;17(2):216–32. doi: 10.1074/mcp.RA117.000470 (PMC5795388; doi:10.1074/mcp.RA117.000470)
Supplement: Supplemental Data [file supp_RA117.000470_133922_0_supp_23978_fzffwf.zip › spectra_annotation/mito_spectra_annotation/105-1-11-1-16-1.pdf]

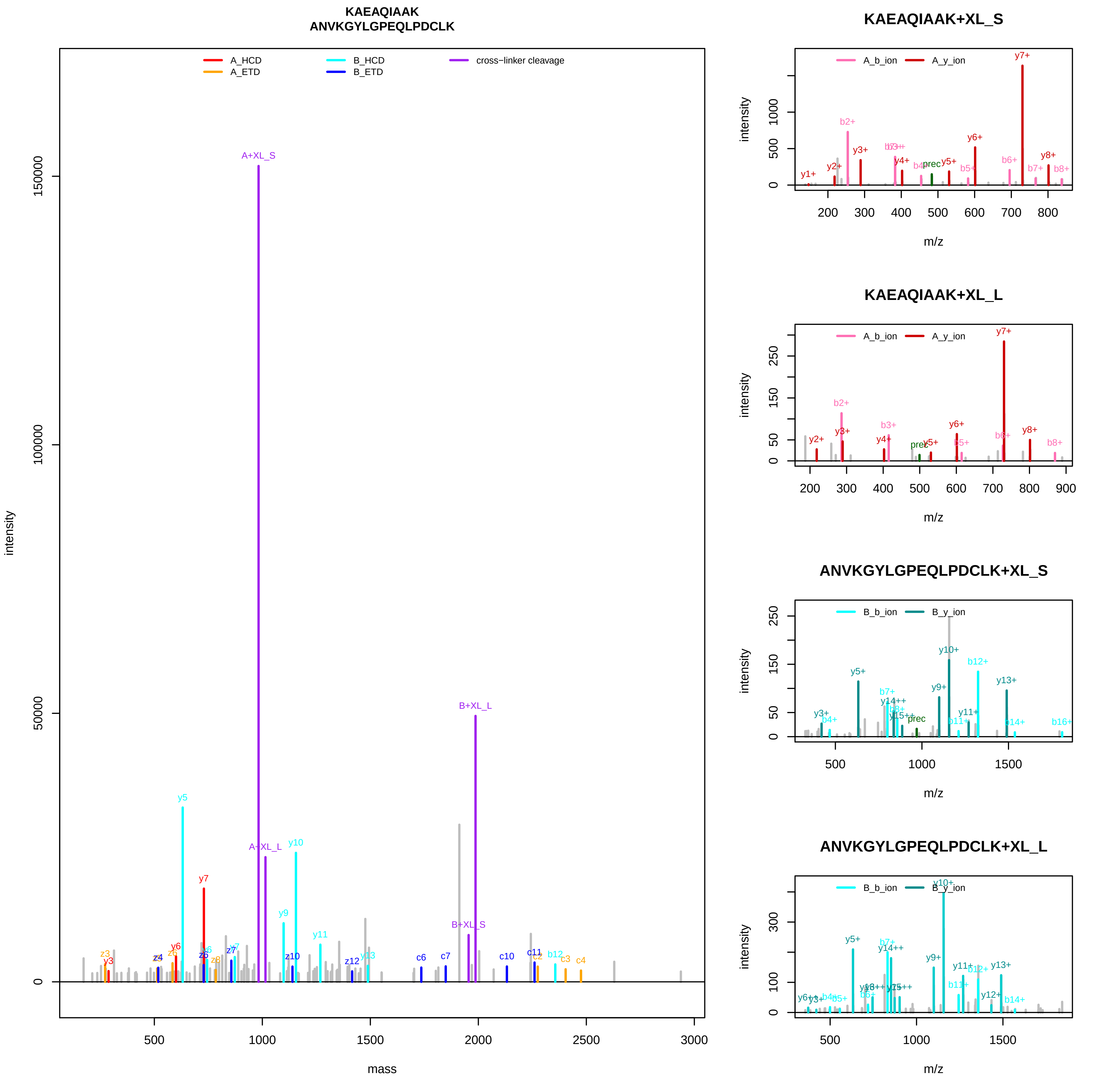

KAEAQIAAK
ANVKGYLGPEQLPDCLK
A_HCD
A_ETD
B_HCD
B_ETD
cross−linker cleavage
intensity
mass
A+XL_S
A+XL_L
B+XL_L
B+XL_S
y5
y7
y6
y3
z3
z4
z6
z7
z10
z12
y9
y10
y11
y13
c6
c7
c10
c11
c2
b12
c3
c4
KAEAQIAAK+XL_S
A_b_ion
A_y_ion
m/z
y1+
y2+
b2+
y3+
y4+
prec
y5+
b5+
y6+
b6+
y7+
b7+
y8+
b8+
KAEAQIAAK+XL_L
ANVKGYLGPEQLPDCLK+XL_S
B_b_ion
B_y_ion
ANVKGYLGPEQLPDCLK+XL_L
